# Uniparental Disomy Leading to a Rare Juvenile Form of ALS

**DOI:** 10.26502/jppch.74050049

**Published:** 2020-10-10

**Authors:** Yigit Karasozen, Kazim A Sheikh, Pedro Mancias, Thy P Nguyen

**Affiliations:** 1 Department of Neurology, McGovern Medical School, University of Texas Houston Health Science Center, Houston, Texas, USA; 2 Department of Pediatrics, McGovern Medical School, University of Texas Houston Health Science Center, Houston, Texas, USA

**Keywords:** Juvenile amyotrophic lateral sclerosis, SIGMAR1, Genomic DNA

## 1. Introduction

Juvenile amyotrophic lateral sclerosis (JALS) is an ultra-rare condition characterized by progressive upper and lower motor neuron weakness before the age of 25 [1, 2]. Incidence is estimated at 1 per million. A subset of genetic etiologies of JALS are autosomal recessive, including SIGMAR1 variants [3]. We report a patient with SIGMAR1 juvenile ALS due to uniparental disomy.

## 2. Case Presentation

Our patient is a 27 year old woman of Nicaraguan descent, who presented to to an orthopedist for toe walking at age 5. She was born to a 31 year old G2P2 mother via vaginal delivery after a full term, uncomplicated gestation. She met all early milestones for motor, language, cognitive, and social development until she started to show symptoms at the age of 4. Over the years, she developed progressive symptoms of muscle weakness with significant atrophy involving her distal arms and legs with development of bilateral pes cavus deformities, foot drop and significant intrinsic hand weakness.

Prior workup included normal routine CSF studies, vitamin levels, plasma amino acids, urine organic acids, very long chain fatty acids, as well as multiple negative genetic panels for spinocerebellar ataxia, Charcot-Marie-Tooth disease, spinal muscular atrophy, and hereditary spastic paraplegia. MRIs of brain and cervical spine were unremarkable. Nerve conduction studies performed at age 12 showed normal sensory responses in sural, median, ulnar, and radial nerves. Compound muscle action potentials had moderate to severely reduced amplitudes, consistent with axonal loss. Needle examination was normal in proximal muscles and showed increased insertional activity, fibrillations and large, polyphasic motor unit action potentials consistent with acute denervation and neurogenic changes in distal muscles. She underwent vastus lateralis muscle biopsy that showed group atrophy with severe type II fiber predominance and scattered esterase positive small angular fibers.

On neurologic examination, mental status and cognitive function were normal. Cranial nerve examination was normal, including normal tongue mass and no fasciculations. Muscle mass was normal proximally, but there was significant atrophy in intrinsic hand muscles, calves and feet. She had a fine postural tremor. Muscle strength was remarkable for 3/5 strength in intrinsic hand muscles, 0/5 dorsiflexion, 3/5 plantar flexion and was otherwise mildly reduced in all other muscle groups. Sensory examination was normal to all modalities. Patient had hyperactive deep tendon reflexes, sustained ankle clonus and extensor plantar reflexes in bilateral lower extremities. Cerebellar examination did not show any evidence of ataxia or dysmetria. Her gait was remarkable for wide base and high steppage due to bilateral foot drop.

At age 19, she had trouble opening jars, but was still able to text on her phone and write. She was ambulating without assistance despite bilateral foot drop, requiring ankle foot orthosis braces. In the subsequent 8 years, she gradually declined in her upper extremity function. She was no longer able to write, and was thus unable to work. She was unable to cut her food, but was still able to feed herself. She developed weakness of her chewing muscles that required her to eat slowly. She did not have significant dysphagia or dysarthria. Her pulmonary function tests were normal. Patient’s parents and brother did not have any neurologic symptoms. She had three children over the years, who are asymptomatic.

At age 19, she elected to undergo whole exome sequencing (WES), which was performed in Baylor College of Medicine (BCM). Genomic DNA was obtained from patient’s whole blood. The exome capture and enrichment was performed with Nimblegen reagents using a custom-designed capture reagent VCRome 2.1, developed by BCM. Massively parallel sequencing was performed on Illumina HiSeq 2000 platform for 100 base paired-end reads. Resulting reads were aligned with the Burrows-Wheeler Aligner. Variant calling was performed using Atlas tools developed in-house by BCM. As a quality control measure patient’s DNA was also analyzed by a SNP-array (Illumina HumanExome-12v1 array) and data was compared with the WES data for >99% concordance. All variants were confirmed with additional Sanger sequencing.

Whole exome sequencing revealed a novel homozygous c.283dupC (p.L95fs) mutation in *SIGMAR1* gene. This mutation does not exist in population data from 114.696 individuals in gnomAD database without any neurologic symptoms [4]. Genomic DNA from the patient’s biological parents’ whole blood samples were analyzed with Sanger sequencing of the targeted region of *SIGMAR1* gene to confirm pathogenicity of the detected variant. Sequence analysis showed that patient’s mother did not carry the variant and her father was heterozygous. Further analysis of the SNP-array data from the patient showed complete absence of heterozygosity for chromosome 9, where the SIGMAR1 gene is located, confirming paternal uniparental disomy of chromosome 9 as the cause of presumed homozygosity in our patient.

## 3. Discussion

Our patient’s case has been previously reported [5]. We sought to illustrate the unique genetic inheritance of her homozygous mutation and discuss the course of her case. Mutations in SIGMAR1 gene cause juvenile amyotrophic lateral sclerosis 16 with autosomal recessive inheritance pattern [1, 4]. Juvenile amyotrophic lateral sclerosis (JALS) is an ultra-rare form of motor neuron disease characterized by upper and lower motor neuron degeneration causing (facial muscle spasticity, spastic dysarthria, and spastic gait) with onset of symptoms before age 25. Usually, onset is in early childhood with a mean age of 6.5 years and reported range of 1 to 20 years [1, 3]. Mutations in several genes including *ALS2*, *SIGMAR1*, *SPG11*, *SETX*, *SOD1*, *UBQLN2*, *TARDB*, *BICD2*, *DDHD1, FUS, CLEC4C, ERLIN1* and *SYNE1* were described to cause JALS [1–3, 6, 7]. Some of these genes were exclusively implicated in juvenile forms of ALS, such as *SIGMAR1*. Most JALS cases are more slowly progressive than adult-onset forms, although there have been a few case reports with unexpected rapid progression. Patients are reported to be bed-ridden by age 12 to 50 [8].

Sigma non-opioid intracellular receptor 1 (*SIGMAR1*) is a 4 exon gene located on chromosome 9p13.3. It encodes an endoplasmic reticulum (ER) chaperone that is highly expressed in spinal motor neurons [9]. This protein is localized to the mitochondrial-associated membranes and involved in lipid metabolism, ER stress response, autophagy initiation, calcium metabolism, and chaperone activity, all of which have a role in neurodegeneration [1, 5, 9]. SIGMAR1 levels were found to be reduced in the spinal cord of patients with ALS [2]. Sigmar1 knockout mice show muscle weakness, axonal degeneration and motor neuron loss [9, 10]. Moreover, in primary motor neuron cultures, inactivation of SIGMAR1 causes dissociation of mitochondria and ER, deregulation of calcium hemostasis, mitochondrial dysfunction, and neurodegeneration [10].

JALS cases are far more likely to be familial compared to adult onset motor neuron disease. Most cases show autosomal recessive inheritance and are frequently reported in consanguineous families. Here we report a non-familial case due to a genetic cause. Currently, the role of uniparental disomy is unclear in ALS and this may be a mechanism behind seemingly sporadic disease. Genetic advances in ALS allow understanding of pathologic processes that lead to motor neuron degeneration. In the future, understanding these mechanisms may lead to future treatments.
